# P-1232. Dose selection of obeldesivir for clinical evaluation in treatment of adult participants with respiratory syncytial virus infection

**DOI:** 10.1093/ofid/ofaf695.1424

**Published:** 2026-01-11

**Authors:** Elham Amini, Eric Salgado, Vincent Chang, Santosh Davies, Darius Babusis, John P Bilello, Olivia Fu, Robert H Hyland, Luzelena Caro

**Affiliations:** Gilead Sciences, Inc., Foster City, CA; Gilead Sciences, Inc., Foster City, CA; Gilead Sciences, Inc., Foster City, CA; Gilead Sciences, Inc., Foster City, CA; Gilead Sciences, Inc., Foster City, CA; Gilead Sciences, Inc., Foster City, CA; Gilead Sciences, Inc., Foster City, CA; Gilead Sciences, Inc., Foster City, CA; Gilead Sciences, Inc., Foster City, CA

## Abstract

**Background:**

Obeldesivir (ODV) is an oral prodrug of the parent nucleoside, GS-441524, which is then converted intracellularly to the active metabolite. ODV is efficacious against respiratory syncytial virus (RSV), SARS-CoV-2, and filoviruses in nonhuman primates (NHP). The pharmacokinetics (PK) and safety of ODV were evaluated in a robust clinical development program (5 Phase 1 trials in healthy participants; 2 Phase 3 trials in patients with COVID-19). Here we describe ODV dose selection for a Phase 2 study in nonhospitalized adults with acute RSV infection, leveraging PK, safety, and efficacy data.

**Methods:**

The optimal RSV ODV dose was chosen to ensure safe and efficacious GS-441524 exposures. The RSV clinical PK efficacy target was based on NHP PK and efficacy studies, wherein administration of ODV 30 and 90 mg/kg/day for 6 days, respectively, had RSV antiviral efficacy. The target plasma GS-441524 maximum concentration and overall exposures associated with NHP efficacious regimens were estimated by extrapolating single-dose PK to multiple-dose PK via nonparametric superposition. Clinical data were used to develop a GS-441524 population PK model characterizing patient subpopulation PK. A logistic regression PK/pharmacodynamic (PD) model characterized the relationship between ODV PK and Grade ≥3 creatinine clearance (CrCL) treatment-emergent laboratory abnormalities (TELAs); however, these TELAs may have been confounded by COVID-19 or pre-existing renal impairment (RI), RI increasing ODV PK, and the low incidence of Grade ≥3 CrCL TELAs. Assuming RSV disease increases GS-441524 exposures by 25% compared to healthy participants, PK and PK/PD were used to predict exposures and rates of CrCL TELAs associated with various dosing regimens.

**Results:**

Based on predicted exposures and CrCL TELAs for those aged >16 years with normal renal function (eGFR ≥90 mL/min/1.73 m^2^) or mild RI (eGFR 60-89 mL/min/1.73 m^2^), an ODV regimen of 700 mg BID on Day 1 and 350 mg BID on Days 2-5 is anticipated to exceed the NHP PK efficacy target GS-441524 exposure and result in an acceptable rate of CrCL TELAs.

**Conclusion:**

To provide an optimal benefit-risk profile, an ODV regimen was selected for the Phase 2 RSV adult study based on the totality of clinical and preclinical data and model-predicted PK and CrCL TELAs.

**Disclosures:**

Elham Amini, PharmD, PhD, Gilead Sciences, Inc.: Employee|Gilead Sciences, Inc.: Stocks/Bonds (Public Company) Eric Salgado, PhD, Gilead Sciences, Inc.: Employee|Gilead Sciences, Inc.: Stocks/Bonds (Public Company) Vincent Chang, PhD, Gilead Sciences, Inc.: Employee|Gilead Sciences, Inc.: Stocks/Bonds (Public Company) Santosh Davies, MD, Gilead Sciences, Inc.: Employee|Gilead Sciences, Inc.: Stocks/Bonds (Public Company) Darius Babusis, BA, Gilead Sciences, Inc.: Employee|Gilead Sciences, Inc.: Stocks/Bonds (Public Company) John P. Bilello, PhD, Gilead Sciences, Inc.: Employee|Gilead Sciences, Inc.: Stocks/Bonds (Public Company) Olivia Fu, MD, Gilead Sciences, Inc.: Employee|Gilead Sciences, Inc.: Stocks/Bonds (Public Company) Robert H. Hyland, DPhil, Gilead Sciences, Inc.: Employee|Gilead Sciences, Inc.: Stocks/Bonds (Public Company) Luzelena Caro, PhD, Gilead Sciences, Inc.: Employee|Gilead Sciences, Inc.: Stocks/Bonds (Public Company)

